# Concentration-dependent increase in symptoms due to diesel exhaust in a controlled human exposure study

**DOI:** 10.1186/s12989-022-00506-6

**Published:** 2022-11-23

**Authors:** Juma Orach, Christopher Francis Rider, Agnes Che Yan Yuen, Christopher Carlsten

**Affiliations:** Air Pollution Exposure Laboratory, Division of Respiratory Medicine, Department of Medicine, Vancouver Coastal Health Research Institute, The University of British Columbia, Vancouver, BC Canada

**Keywords:** Controlled human exposures, Symptoms, Diesel exhaust, Particulate matter, Airways

## Abstract

**Background:**

Traffic-related air pollution (TRAP) exposure causes adverse effects on wellbeing and quality of life, which can be studied non-invasively using self-reported symptoms. However, little is known about the effects of different TRAP concentrations on symptoms following controlled exposures, where acute responses can be studied with limited confounding. We investigated the concentration–response relationship between diesel exhaust (DE) exposure, as a model TRAP, and self-reported symptoms.

**Methods:**

We recruited 17 healthy non-smokers into a double-blind crossover study where they were exposed to filtered air (FA) and DE standardized to 20, 50, 150 µg/m^3^ PM_2.5_ for 4 h, with a ≥ 4-week washout between exposures. Immediately before, and at 4 h and 24 h from the beginning of the exposure, we administered visual analog scale (VAS) questionnaires and grouped responses into chest, constitutional, eye, neurological, and nasal categories. Additionally, we assessed how the symptom response was related to exposure perception and airway function.

**Results:**

An increase in DE concentration raised total (β ± standard error = 0.05 ± 0.03, *P* = 0.04), constitutional (0.01 ± 0.01, *P* = 0.03) and eye (0.02 ± 0.01, *P* = 0.05) symptoms at 4 h, modified by perception of temperature, noise, and anxiety. These symptoms were also correlated with airway inflammation. Compared to FA, symptoms were significantly increased at 150 µg/m^3^ for the total (8.45 ± 3.92, *P* = 0.04) and eye (3.18 ± 1.55, *P* = 0.05) categories, with trends towards higher values in the constitutional (1.49 ± 0.86, *P* = 0.09) and nasal (1.71 ± 0.96, *P* = 0.08) categories.

**Conclusion:**

DE exposure induced a concentration-dependent increase in symptoms, primarily in the eyes and body, that was modified by environmental perception. These observations emphasize the inflammatory and sensory effects of TRAP, with a potential threshold below 150 µg/m^3^ PM_2.5_. We demonstrate VAS questionnaires as a useful tool for health monitoring and provide insight into the TRAP concentration–response at exposure levels relevant to public health policy.

**Supplementary Information:**

The online version contains supplementary material available at 10.1186/s12989-022-00506-6.

## Introduction

Traffic-related air pollution (TRAP) exposure causes adverse health effects and is a risk factor for morbidity worldwide [1–3]. The effects of pollution on subjective wellbeing and quality of life [4], in particular, can be assessed non-invasively using self-reported symptoms questionnaires [5–7]. Symptoms are also robust indicators of the pollution-associated exacerbation of cardiopulmonary diseases [6, 8–10] and are correlated with other clinical health measures, such as fractional exhaled nitric oxide (FeNO) [11] and forced expiratory volume in 1 s (FEV_1_) [12, 13].

Diesel exhaust (DE) consists of gases and particulate matter (PM), including a particularly harmful fraction with a diameter < 2.5 µm (PM_2.5_), that interacts with cells at mucosal surfaces to instigate inflammation, oxidative stress, epithelial damage and sensory nerve activation [1, 14]. In addition to the systemic response mobilized by these interactions, PM with diameter < 0.1 µm (PM_0.1_) may directly translocate into the blood to propagate systemic inflammation [15, 16]. Additional roles have been proposed for psychological factors, such as exposure perception [17], in affecting pollution-associated symptoms.

Concentration–response (C–R) relationships help elucidate the link between exposures and effects and have been used to investigate symptom responses over a broad range of TRAP concentrations in epidemiological studies [9, 10, 18, 19]. In controlled human exposure (CHE) studies, where residual confounding is limited, the acute effects of TRAP exposure on symptoms have also been studied, commonly using diesel exhaust (DE) as a model of TRAP [20–24]. However, the C–R relationship between TRAP exposure and symptoms is relatively unexplored in CHE studies [25]. An improved understanding of the link between air pollution and symptoms, and the role of perception in this relationship, is crucial in evaluating the impacts of air pollution on wellbeing.

In this study, we investigated the C–R relationship for self-reported symptoms after controlled human exposures. We hypothesized that higher DE concentrations would increase symptoms. Additionally, we studied the role of environmental perception in this C–R relationship. Lastly, we investigated the relationship between symptoms and clinical measures of airway function and inflammation. We report concentration-dependent effects of TRAP on symptoms that could inform future strategies to assess the impacts of air exposure non-invasively.

These results have previously been reported in the form of a conference abstract [26].

## Methods

### Controlled diesel exhaust exposures

The Diesel Induces Concentration-dependent Effects (DICE) study (NCT03234790) was a double-blind crossover study approved by the University of British Columbia Research Ethics Board (H16-03053). Healthy non-smokers aged 19–49 were recruited using referrals and online advertisements. Following informed consent, participants were screened for respiratory and cardiac abnormalities by the study physician. Participants were included if they were healthy, aged 19–49, non-smokers, and able to communicate and complete study procedures. Exclusion criteria included pregnancy/breast-feeding, conflicting time commitments and inhaled corticosteroid use. Before each study visit participants completed a standard common cold questionnaire to confirm that they did not have upper respiratory tract infection symptoms and were asked to withhold caffeine and bronchodilator use. Visits were postponed by at least 4 weeks if a possible respiratory infection was reported. Participants were exposed to filtered air (FA) and DE standardized to 20, 50, 150 µg/m^3^ PM_2.5_ over four separate visits at the Air Pollution Exposure Laboratory [27]. These PM_2.5_ concentrations are common in DE exposure studies [28], and approximate real world urban [29] and occupational [30] levels. Exposures were completed in randomized orders with each separated by a ≥ 4-week washout period. In the event of substantial spikes in ambient air pollution, exposures were postponed by at least 4 weeks. DE was generated by an EPA Tier 3-compliant, 6.0 kW Coliseum GY6000 generator, with a 406 cc Yanmar L 100 EE 4-stroke diesel engine with a constant 2.5 kW load, which upon failure in February 2021, was replaced by a 4.5 kW 1B30E Hatz EPA/Euro-Stage Tier 5-compliant engine (for all exposures for participants 14–17) to reflect contemporary technology. Exposure details for the diesel engines are presented in Additional file 1: Table S1. During the exposures, participants exercised intermittently on a stationary bike for 15 min/h at a power-to-weight ratio estimated to achieve a ventilation rate of 15 L/min/m^2^.

### Questionnaires

Symptoms typically associated with air pollution exposure in the literature [17, 24, 31] were evaluated by the participants using a visual analog scale (VAS) [32, 33] questionnaire (Additional File 1: Figure S1) pre-exposure, 4 h and 24 h post-exposure. To assess exposure perception, participants responded to a VAS questionnaire about the environment in the exposure booth and were asked if they thought their exposure was to FA or DE.

### Spirometry

Lung function was measured by spirometry before, and at 4 h and 24 h from the start of the exposure according to American Thoracic Society/European Respiratory Society guidelines [34]. Airway responsiveness was measured by the methacholine response before and at 24 h from the start of the exposure using the 2-min tidal breathing technique [35]. Novo-Salbutamol HFA (TEVA; ON, CA) was administered following the baseline methacholine challenge to restore lung function. Methacholine provocation concentration to cause a 20% drop in FEV_1_ (PC_20_) was estimated using the appropriate equations [36, 37].

### FeNO

FeNO was measured using a NIOX VERO® machine (NIOX, ON, CA) machine before and at 4 h and 24 h from the start of exposures according to American Thoracic Society/European Respiratory Society guidelines [38].

### Statistical analyses

Measurements of VAS questionnaires were completed by at least 2 technicians independently, entered into REDCAP 10.4.0 (© 2021 Vanderbilt University) and checked for consistency and accuracy using in-built REDCAP tools. Baseline values for all outcomes were subtracted from values at subsequent timepoints to obtain delta values. To limit the penalty for multiple comparisons, symptoms were analyzed at the category level (summarized in Table 1) similar to Carlsten et al*.* [17].

**Table 1:** Symptom questions and categories

Symptom category	Question
Chest	Please rate your level of throat irritation
	Do you feel a need to cough?
	Do you suffer from shortness of breath right now?
	Are you currently experiencing a whistling or hissing sound while breathing?
	Are you experiencing chest pain?
Constitutional	Is your skin itchy or dry?
	Are you sweating right now?
	Do you have a fever right now?
	How would you rate your general wellbeing?
Eye	Do your eyes itch or sting?
	Please rate how dry your eyes are right now
	Are your eyes running or watering?
Neurological	Do you feel sleepy or drowsy?
	Do you have a headache right now?
	Do you find it difficult to concentrate?
	Are you currently feeling nauseous?
Nose	Does your nose feel irritated, itchy, stinging or dry?
	Do you currently have a runny nose?
	Is your nose blocked right now?

Symptom scores (standardized to a 10 cm scale) were summed within each category and also as a total including all categories for analysis

Linear mixed effects models, with a participant-specific intercept to adjust for repeated measures, were used to assess the effects of DE exposure on symptoms (package nlme_3.1-157). To estimate the C–R, a model (1) of symptom category delta values and PM_2.5_ was fitted, while in model (2) symptom category delta values and controlled exposure condition groups were fitted to identify potential effects thresholds. To evaluate the effect of perception on symptoms from DE exposure, a model (3) was fit with participant’s perception of environment/exposure as a modifier of the relationship between symptoms and PM_2.5_.

- $${Y}_{i,j}={\beta }_{1}\left({PM}_{2.5}\right)+{\beta }_{0}+{\mu }_{i}+{\varepsilon }_{i,j}$$
- $${Y}_{i,j}={\beta }_{1}\left(exposure\, condition\, group\right)+{\beta }_{0}+{\mu }_{i}+{\varepsilon }_{i,j}$$
- $${Y}_{i,j}={\beta }_{1}\left({PM}_{2.5}\right)* perception+{\beta }_{0}+{\mu }_{i}+{\varepsilon }_{i,j}$$

where i = *i*th individual, j = *j*th repeated measurement, β_1_ = slope, β_0_ = overall intercept, µ = participant intercept, ε = error term.

Model assumptions were checked and where appropriate, data were log-transformed. Correlations between outcomes were calculated using repeated measures correlations (rmcorr package V.0.4.5). All statistical analysis was performed using R version 4.2.0. *P* ≤ 0.05 were considered statistically significant, while *P* values 0.051–0.1 were considered to be “trending towards significance”.

## Results

### Study population

Of the 20 participants enrolled in the study, 15 completed all exposures and were included in the analysis. Additionally, 2 participants who did not complete one of four exposures (one in each of the DE50 and DE150 categories) were included in the analysis to give a total of 17 participants (Table 2).

**Table 2:** A summary of participant demographics

Characteristics	Mean (95% CI)
Sex (male/female)	9/8
Age	28.18 (23.89, 32.46)
BMI	24.74 (22.52, 26.95)
FEV_1_	3.64 (3.25, 4.04)
FEV_1_%predicted	98.88 (94.61, 103.16)
FVC	4.49 (3.99, 4.99)
FEV_1_: FVC	0.82 (0.78, 0.85)
FeNO (ppb)	23.29 (15.66, 30.93)
PC_20_	> 128 mg/mL

Age, BMI and lung function measures were recorded at the screening visit. Methacholine FeNO and PC_20_, which were not recorded at the screening visit, are based on average pre-exposure values for all the exposures. PC_20_ > 16 are considered normally responsive according to the European Respiratory Society Technical Standard

BMI, body-mass index; FeNO, fractional exhaled nitric oxide; FEV_1_, forced expiratory volume in 1 s; FVC, forced vital capacity; PC_20_, methacholine provocation concentration causing a 20% drop in FEV_1_

The remaining 3 participants withdrew from the study due to scheduling constraints. Details are summarized in flow diagram in Additional file 1: Figure S2.

### Exposure characteristics

Exposure data are presented by nominal PM_2.5_ exposure group and diesel engine in Additional file 1: Table S1. While there were differences between the engines across some measures (most notably more ultrafine particles and hence total particles with the newer Tier 5 engine, at similar levels of PM_2.5_, as expected given the older engine was subject to combustion inefficiency over years of operation), engine type did not significantly modify symptom responses (Additional file 1: Table S2).

### DE induced a concentration-dependent increase in total, eye, and constitutional symptoms

**Fig. 1:**
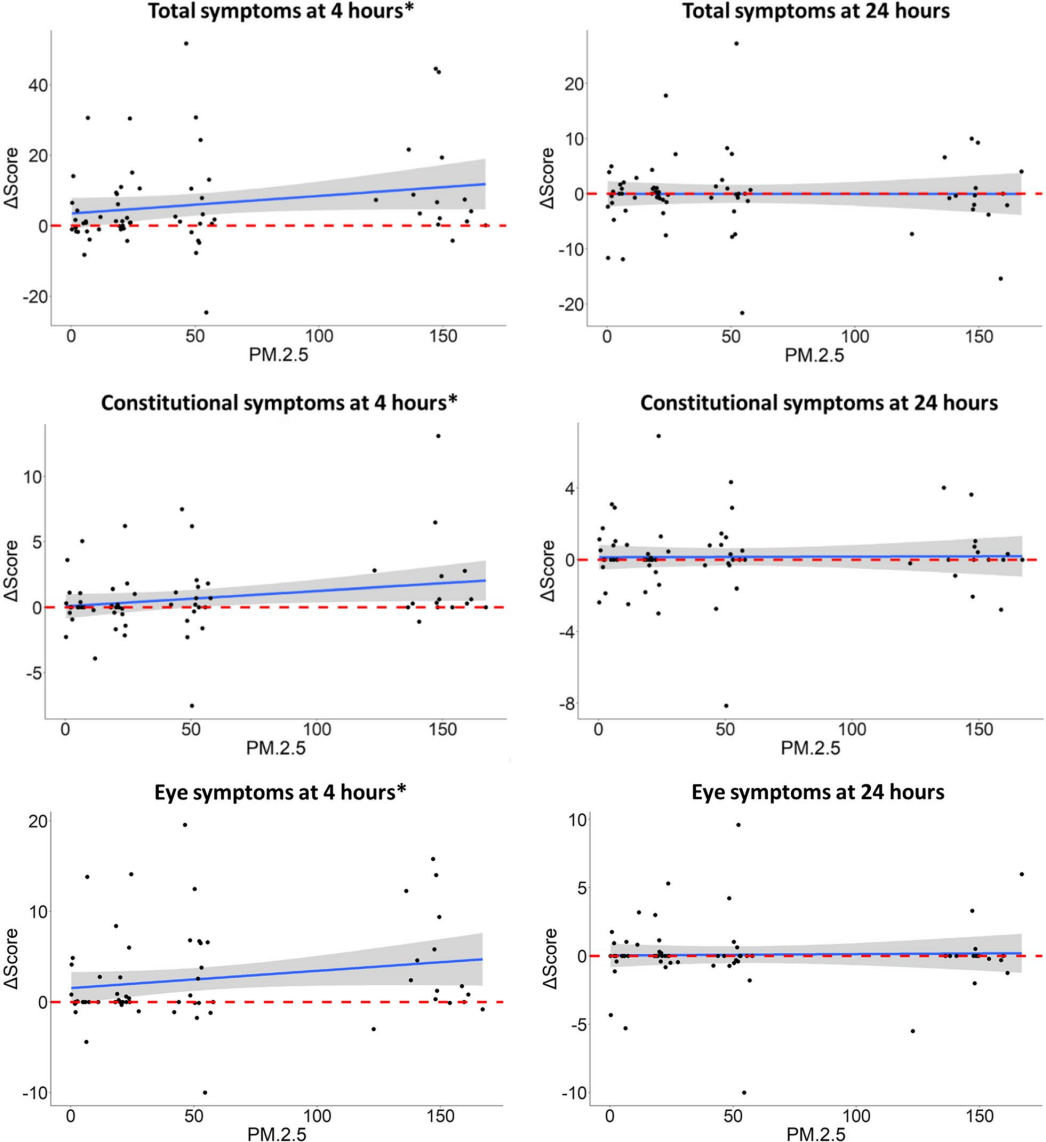

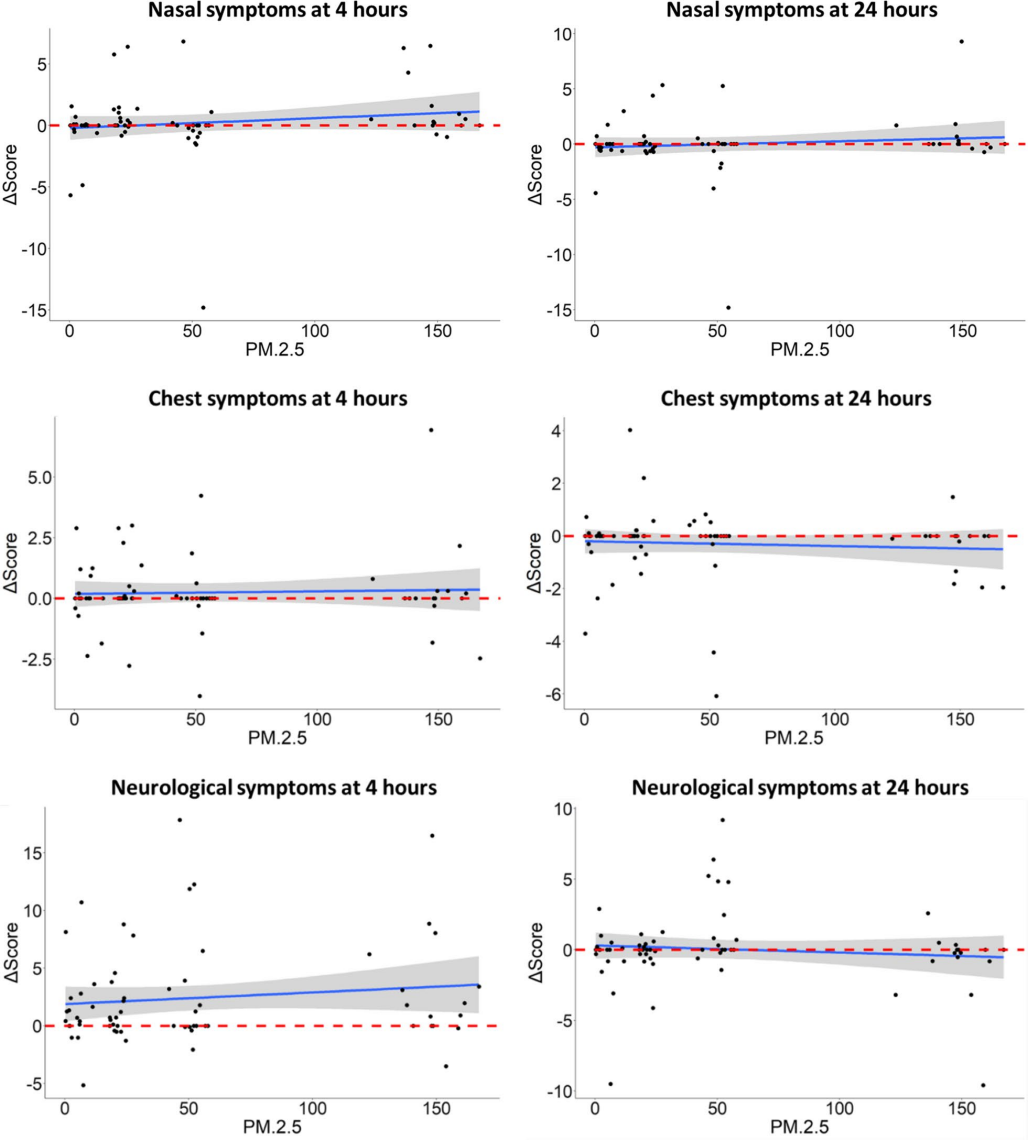
Diesel exhaust (DE) concentration–response for symptom categories. Symptoms were recorded before, and at 4 and 24 h after the start of exposures to filtered air and diesel exhaust (DE) standardized to 20, 50 and 150 µg/m^3^ PM_2.5_. X axes show change in symptom scores from baseline, while Y axes show PM_2.5_ concentrations (µg/m^3^). Shaded grey regions represent 95% confidence intervals, and the horizontal dashed lines represent 0 (no change from baseline). Linear mixed effects models were fitted with participant ID as a random effect: *P ≤ 0.05

At 4 h post-exposure, DE induced a concentration-dependent increase in total symptoms (β ± standard error = 0.05 ± 0.03, *P* = 0.04), driven by the constitutional (0.01 ± 0.01, *P* = 0.03) and eye (0.02 ± 0.01, *P* = 0.05) symptom categories (Fig. 1). Of the underlying questions, participants primarily reported itching and stinging in the eyes (*P* = 0.03) and itchiness or dryness of the skin (*P* = 0.06).

Redoing this analysis with only the 15 participants that completed all four exposures did little to change the output, but eye category symptoms were no longer significantly changed (*P* > 0.1) and hence total symptoms moved to borderline significance (*P* = 0.05–0.1). However, this analysis is adversely affected by outliers in the smaller dataset.

**Fig. 2:**
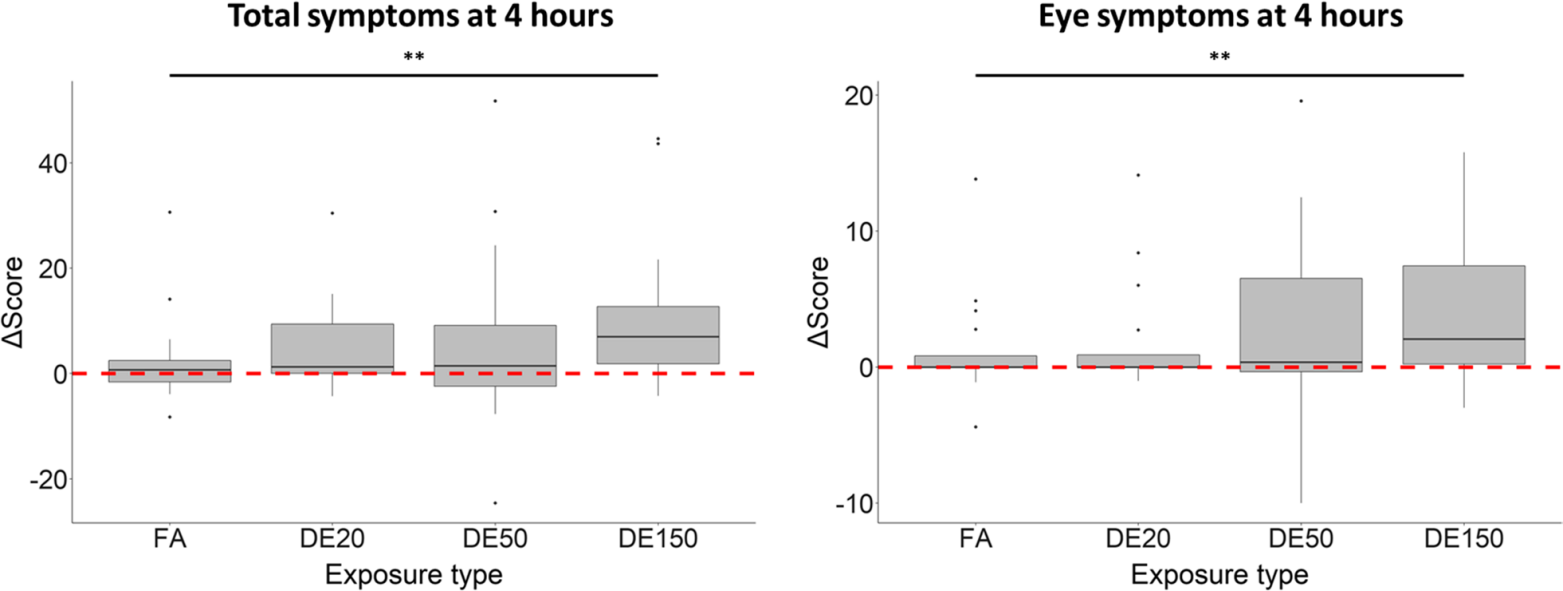
Effects of diesel exhaust (DE) on symptom categories by exposure group. Symptoms were recorded before, and at 4 and 24 h after the start of exposures to filtered air and DE standardized to 20, 50 and 150 µg/m^3^ PM_2.5_. X axes show represent change in symptom scores from baseline; Y axes show nominal exposure conditions. Horizontal dashed lines represent 0 (no change from baseline). Linear mixed effects models were fitted with participant ID as a random effect: **P ≤ 0.05, *P = 0.051–0.1

Compared to FA, DE at 150 µg/m^3^ induced an increase in total (8.45 ± 3.92, *P* = 0.04) and eye (3.18 ± 1.55, *P* = 0.05) symptoms, and a trend towards significance in the constitutional (1.49 ± 0.86, *P* = 0.09) and nasal (1.71 ± 0.96, *P* = 0.08) symptoms (Fig. 2). These effects were all absent at 24 h.

### The symptom concentration–response was modified by environmental perception

Increasing perception of noise (− 0.07 ± 0.03, *P* = 0.01) and temperature (− 0.06 ± 0.03, *P* = 0.02) attenuated the concentration-dependent increase in total symptoms, driven by effects in both the eye (noise effect = − 0.02 ± 0.01, *P* = 0.02; temperature effect = − 0.03 ± 0.01, *P* = 0.01) and constitutional categories (noise effect = − 0.01 ± 0.01, *P* = 0.03) (Table 3). Anxiety enhanced the concentration-dependent increase in constitutional symptoms (0.01 ± 0.00, *P* = 0.03).

**Table 3:** Concentration–response effect modification by environmental perception

Symptom category (at 4 h)	Perception measure	β (SE)	*P* value
Total	Lighting	− 0.02 (0.03)	0.44
	Glare	− 0.01 (0.01)	0.29
	Noise	− **0.07 (0.03)**	**0.01**
	Temperature	− **0.06 (0.03)**	**0.02**
	Humidity	− 0.03 (0.05)	0.50
	Air circulation	0.03 (0.03)	0.30
	Air quality	− 0.03 (0.04)	0.52
	Odor	0.03 (0.03)	0.23
	Ventilation	0.00 (0.01)	0.72
	Anxiety	0.02 (0.02)	0.38
Constitutional	Lighting	− 0.01 (0.01)	0.08
	Glare	0.00 (0.00)	0.50
	Noise	− 0.01 (0.01)	0.06
	Temperature	− **0.01 (0.01)**	**0.03**
	Humidity	0.00 (0.01)	0.96
	Air circulation	0.01 (0.01)	0.32
	Air quality	0.01 (0.01)	0.34
	Odor	0.01 (0.01)	0.09
	Ventilation	0.00 (0.00)	0.55
	Anxiety	**0.01 (0.00)**	**0.03**
Eyes	Lighting	0.01 (0.01)	0.46
	Glare	− 0.01 (0.00)	0.23
	Noise	− **0.02 (0.01)**	**0.02**
	Temperature	− **0.03 (0.01)**	**0.01**
	Humidity	0.00 (0.02)	0.91
	Air circulation	0.01 (0.01)	0.19
	Air quality	− 0.03 (0.02)	0.13
	Odor	0.01 (0.01)	0.27
	Ventilation	0.00 (0.00)	0.60
	Anxiety	0.00 (0.01)	0.94

Symptom scores were summed into categories

Significant effects (*P* < 0.05) are bolded

**Fig. 3:**
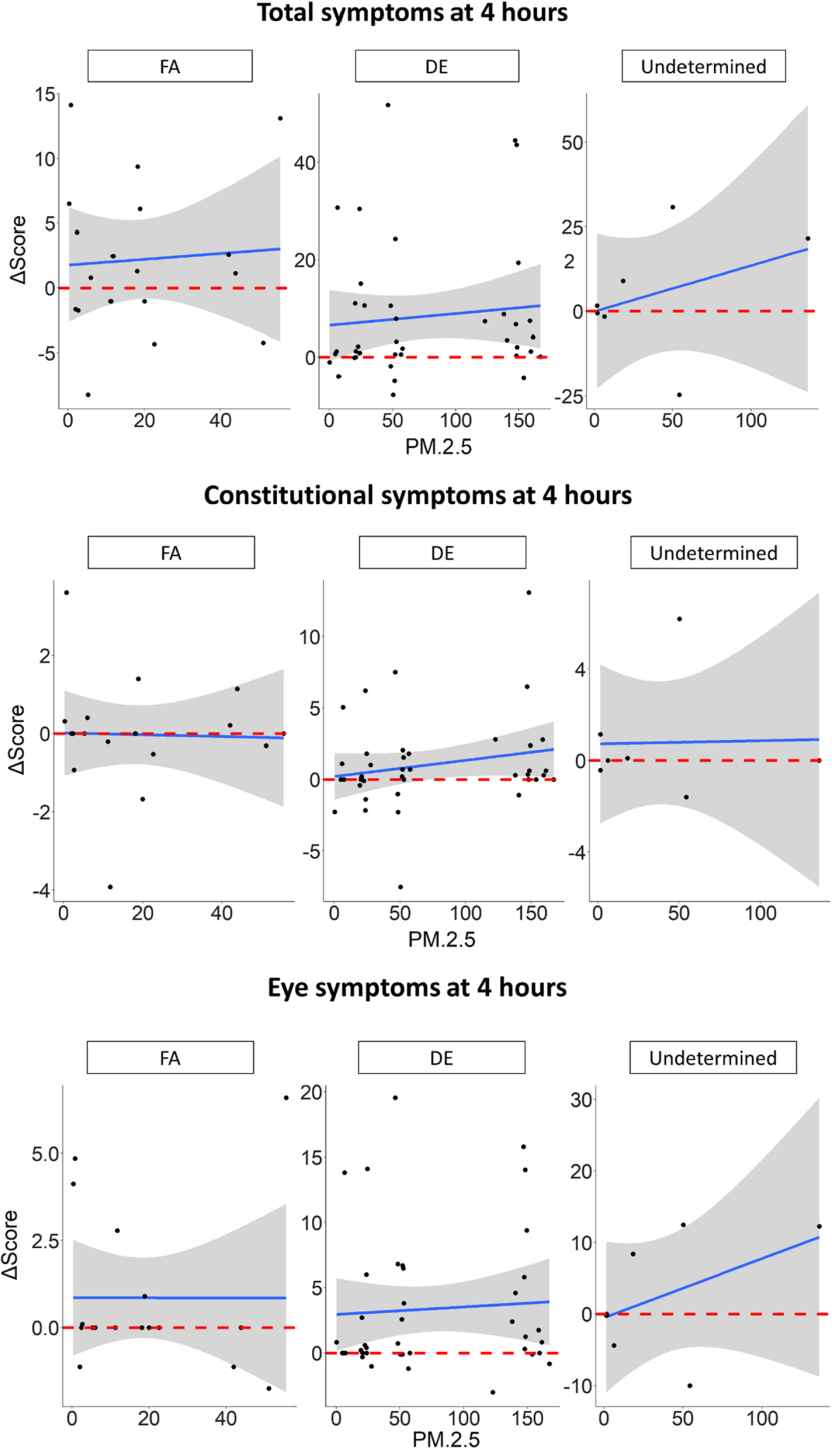
Effect of perception on the concentration–response between diesel exhaust (DE) and symptoms. Symptoms data was recorded before, and at 4 and 24 h after the start of exposures to filtered air and DE standardized to 20, 50 and 150 µg/m^3^ PM_2.5_. Y axes show change in symptom scores from baseline; X axes show PM_2.5_ concentrations (µg/m^3^). Shaded grey regions represent 95% confidence intervals, and the horizontal dashed lines represent 0 (no change from baseline). Linear mixed effects models were fitted with perceived exposure condition as an interaction term and participant ID as a random effect

However, symptom responses were not modified by participants perception of whether they were exposed to DE or FA (Fig. 3). Participant sex did not modify symptoms (data not shown).

### The symptom concentration–response was correlated with airway inflammation

The concentration–response for total symptoms was moderately positively correlated with ΔFeNO at 4 h (r = 0.29 ± 0.13, *P* = 0.04) and 24 h (r = 0.39 ± 0.12, *P* < 0.00) driven by effects in the constitutional and eye categories (details summarized in Table 4). Symptoms were not correlated with methacholine PC_20_ and FEV_1_.

**Table 4:** Repeated measures correlations between change in symptoms and airway function measures

Symptom category (at 4 h)	Correlate	Timepoint (h)	r (std error)	*P* value
Total	Percentage ΔFeNO	4	**0.29 (0.13)**	**0.04**
		24	**0.39 (0.12)**	** < 0.00**
	Percentage ΔFEV_1_	4	0.04 (0.14)	0.78
		24	0.07 (0.14)	0.64
	Δlog PC_20_	24	− 0.06 (0.15)	0.71
Constitutional	Percentage ΔFeNO	4	0.12 (0.14)	0.41
		24	**0.34 (0.13)**	**0.02**
	Percentage ΔFEV_1_	4	0.04 (0.14)	0.79
		24	0.17 (0.14)	0.24
	Δlog PC_20_	24	− 0.02 (0.15)	0.91
Eyes	Percentage ΔFeNO	4	**0.32 (0.13)**	**0.02**
		24	**0.40 (0.12)**	** < 0.00**
	Percentage ΔFEV_1_	4	0.04 (0.14)	0.78
		24	0.09 (0.14)	0.55
	Δlog PC_20_	24	− 0.07 (0.15)	0.65

Symptom scores were summed into categories

Significant effects (*P* < 0.05) are bolded

## Discussion

Exposure to air pollution is associated with adverse health effects, whose impact on wellbeing and quality of life can be assessed using symptoms [5–7]. Current knowledge on the effects of TRAP on symptoms could be improved by better understanding of their C–R relationship. In this study, we investigated and identified concentration-dependent increases in symptoms that were modified by environmental perception.

We observed a significant concentration-dependent increase in total symptoms driven primary by eye and constitutional symptoms. PM and gases routinely interact with exposed surfaces of the body such as the skin [39, 40] and eyes [41] where they may be inflammatory. In the eyes, these pollutants can cause dryness and irritation [41, 42] through oxidative stress [43], mucin disruption, and loss of microvilli, corneal and goblet cells [14, 44, 45]. In the airways, PM_2.5_ can enter the alveoli where it induces inflammation and oxidative stress that may result in systemic immune mobilization [15, 46]. This “spill over”, in addition to the penetration of PM_0.1_ into the blood stream, may cause adverse neurological, constitutional, and systemic effects. PM_2.5_, the primary surrogate for DE concentration in our analyses, was correlated with other pollutants in the DE mixture. Thus, gases and TVOCs may have a role in the symptom response attributed to PM_2.5_ here, but readers should not infer cause due to any particular aerosol component as this model exposure is a paradigm of traffic-related air pollution with PM_2.5_ simply used as a metric for reasonably standardizing conditions upon a common parameter. The correlation between symptoms and airway inflammation in our study lends credence to inflammation as a potential physiological pathway through which air pollutants cause symptoms. However, the symptom response was unaccompanied by changes in lung function, similar to other acute exposure studies assessing similar endpoints [20, 47]. This absence of changes in lung function after acute air pollution exposure is likely due to resilience to acute low-concentration DE exposures in healthy populations and has been corroborated by other controlled exposure studies [28, 48, 49].

Our findings are consistent with reports of DE-induced eye and constitutional symptoms in other controlled exposure studies [21–24, 50]. Notably, we did not observe any of the neurological or airway symptoms reported by these studies and others [47]. In contrast, other studies, which included rhinitis [51] and metabolic syndrome patients [17], did not report effects on symptoms. Interestingly, Carlsten et al*.* (2013) also reported a prominent role of perceived exposure condition (DE vs FA) in symptoms after DE exposure, albeit not as an effect modifier [17].

The symptom response that we observed was modified by perceived environmental temperature, noise, and anxiety. Higher temperatures and noise levels attenuated the increase in total, eye and constitutional symptoms, while anxiety enhanced constitutional symptoms. Known relationships between air pollution exposure and temperature [52–56], noise [57, 58] and anxiety [59–61] in epidemiological literature are mixed and vary by endpoints. For example, air pollution acts synergistically with and directly on temperature and anxiety respectively in some studies [52, 55, 60], but not others [59, 61]. Notably, these studies investigated ambient (not perceived) temperature and noise, and only explored anxiety as a direct effect of air pollution. In controlled human exposure studies, where ambient temperature and noise are relatively constant, the relationship between air pollution and environmental perception is relatively unexplored. Some studies have investigated the direct effects of air pollution exposure and noise, reporting no significant effect on anxiety symptoms [22] and deleterious effects on other endpoints [62, 63]. Since ambient temperature and noise were consistent throughout our study, the interactions we observed could reflect a delineation between the perceived and true (measured) environment. This “mismatch” could be explained individual or localized psychological and physiological factors like sensitivity, discomfort and annoyance, which independently influence environmental perception and subjective symptoms [64–67]. While the underlying psychological triggers in our experimental setting are unclear, it is possible that a primary feeling, such as discomfort, influenced perception. For example, discomfort associated with anxiety may be responsible for anxiety-related enhancement of symptoms. Similarly, the discomfort due to perceived coldness, which is associated with eye irritation [68], may explain the attenuation of symptoms with increasing perceived temperature. The attenuation of symptoms by increased perceived noise is surprising, considering that others have reported symptom enhancement [64, 65, 69]. Interestingly, overall perceived exposure condition (DE or FA) did not modify symptoms, which indicates effective experimental blinding of symptom responses, but this analysis may be limited by unbalanced comparison groups. Different participant demographics, in addition to methodological differences, such as statistical approaches, endpoints, exposure levels and durations, limit direct comparisons between our work and others. The unknown psychological triggers and relatively small interaction effect sizes, highlight the need for corroboration and further exploration in future studies. Our study is the first DE exposure study to use multiple exposure concentrations to examine the linear C-R relationship between TRAP and symptoms and examine interactions with environmental perception.

The significant increase in symptoms at 150 µg/m^3^ PM_2.5_, compared to FA, is consistent with an effect threshold below 150 µg/m^3^, similar to a threshold below 140 µg/m^3^ of total suspended particles suggested by Mølhave et al.[70]. Moreover, Vilcassim et al*.* reported an increase in symptoms when participants travelled from low (< 35 µg/m^3^ PM_2.5_) to high (> 100 µg/m^3^ PM_2.5_) pollution cities [18], estimating a 40 µg/m^3^ threshold. The resolution of symptoms at 24 h after acute exposure in our study is consistent with recovery after the cessation of air pollution exposure observed by Vilcassim et al*.* and Mølhave et al*.* [18, 70]. The transient nature of symptoms, relatively small effect sizes and absence of a strong relationship with lung function may indicate that these effects are often subclinical in this healthy population. Nevertheless, our findings are important to biological plausibility and may provide useful estimates and potential thresholds for assessing the health impacts of air pollution in a healthy population.

Although the exposure duration in this study is the longest for a controlled DE study to date, it does not fully replicate complex typically day-to-week long real-world exposures [28]. Secondly, our study recruited a relatively small sample of healthy non-smokers and may not be sufficiently generalizable to other populations [71]. Thus, future studies may delve further into susceptibility factors that modify the C-R relationship, as well as physiological mechanisms associated with reported symptoms. Lastly, we report relatively novel findings of perceived environmental modifiers of the air pollution exposure symptom response that warrant replication in future studies.

In this controlled DE exposure study, we detailed a concentration–response relationship between particulate matter and self-reported symptoms, and identified perceived temperature, noise, and anxiety as potential modifiers of this relationship. Our research not only highlights the utility of visual analog scale questionnaires as non-invasive tools for assessing the health effects of air pollution, but also provides effect estimates and modifiers over a range of epidemiologically relevant PM_2.5_ levels. This may be crucial in adopting self-reported questionnaires as non-invasive tools for health monitoring and developing public health guidelines for air pollution.

## Supplementary Information

Additional file 1. Supplemental tables and figures.

## Data Availability

The datasets supporting the conclusions of this article are available from the corresponding author upon reasonable request.
