# Supplementary material for: Concentration-dependent increase in symptoms due to diesel exhaust in a controlled human exposure study
Source: Part Fibre Toxicol. 2022 Nov 23;19:66. doi: 10.1186/s12989-022-00506-6 (PMC9685897; doi:10.1186/s12989-022-00506-6)
Supplement: Supplementary file 1 — Additional file 1. Supplemental tables and figures. [file 12989_2022_506_MOESM1_ESM.docx]

**Concentration-dependent increase in symptoms due to diesel exhaust in a controlled human exposure study**

Juma Orach^1^, Christopher Francis Rider^1^, Agnes Che Yan Yuen^1^, Christopher Carlsten^1^

1. Air Pollution Exposure Laboratory, Division of Respiratory Medicine, Department of Medicine, Vancouver Coastal Health Research Institute, The University of British Columbia, Vancouver, British Columbia, Canada

# **Additional File 1**

**Table S1.** Controlled diesel exhaust exposure measurements summarized by exposure group and diesel engine.

|  | FA (SD) | | 20 (SD) | | 50 (SD) | | 150 (SD) | |
| --- | --- | --- | --- | --- | --- | --- | --- | --- |
| Diesel Engine | Tier 5 | Tier 3 | Tier 5 | Tier 3 | Tier 5 | Tier 3 | Tier 5 | Tier 3 |
| PM_2.5_ (µg/m^3^) | 4 (2) | 4 (4) | 20 (3) | 22 (3) | **56 (3)** | **50 (4)** | 142 (16) | 144 (30) |
| NO (ppb) | 294 (207) | 317 (419) | 581 (51) | 1537 (928) | 1030 (200) | 2335 (1328) | 1910 (606) | 3989 (2421) |
| NO_2_ (ppb) | 22 (22) | 21 (12) | 39 (38) | 87 (110) | 83 (95) | 126 (81) | 97 (103) | 144 (117) |
| NO_x_ (ppb) | 315 (227) | 337 (425) | 618 (59) | 1619 (995) | 1109 (187) | 2454 (1375) | 2004 (580) | 4122 (2446) |
| CO (ppm) | 3 (0) | 2 (1) | **2 (1)** | **4 (2)** | **3 (1)** | **6 (2)** | **3 (1)** | **11 (3)** |
| CO_2_ (ppm) | **706 (22)** | **948 (118)** | 745 (61) | 1754 (1513) | 817 (19) | 2930 (4749) | **958 (92)** | **2438 (1013)** |
| TVOC (ppb) | 176 (34) | 220 (91) | 254 (130) | 282 (128) | 577 (120) | 469 (153) | **1389 (718)** | **830 (267)** |
| Ultrafine particle number concentration (#/cm^3^) | **209478 (47014)** | **22588 (27830)** | **180141 (85908)** | **29061 (19579)** | **211055 (94475)** | **42342 (32323)** | **300124 (175701)** | **130226 (119220)** |
| Total particle number concentration (#/cm^3^) | **317015 (74918)** | **31872 (42309)** | **262936 (130493)** | **41611 (28972)** | **320803 (148174)** | **63804 (47326)** | 458638 (266781) | 216645 (220669) |
| Particle size (nm) | 75 (1) | 64 (25) | 68 (6) | 75 (14) | 71 (3) | 74 (11) | **72 (4)** | **85 (8)** |
| Temperature (^0^C) | 24 (1) | 24 (2) | 23 (2) | 24 (2) | 23 (2) | 25 (2) | 24 (2) | 24 (1) |
| Relative humidity (%) | 38 (7) | 38 (5) | 36 (7) | 38 (8) | 39 (14) | 39 (8) | 36 (19) | 37 (8) |

Abbreviations: CO_2_ = carbon dioxide; CO = carbon monoxide; NO_2_ = nitrogen dioxide; NO = nitrogen oxide; NO_x_ = nitrogen oxides; PM_2.5_ = particulate matter with diameter ≤2.5µm; ppb = parts per billion; ppm = parts per million. TVOC = total volatile organic compounds. ANOVA comparisons with significant differences between the diesel engines (P<0.05) **bolded**.

**Table S2.** Symptom response modification by diesel engine type.

| Symptom category (timepoint) | β (std error) | P value |
| --- | --- | --- |
| Eyes (4h) | -0.04 (0.02) | 0.14 |
| Eyes (24h) | -0.02 (0.01) | 0.13 |
| Nose (4h) | 0.00 (0.02) | 0.91 |
| Nose (24h) | 0.00 (0.01) | 0.89 |
| Chest (4h) | 0.00 (0.01) | 0.82 |
| Chest (24h) | 0.00 (0.01) | 0.75 |
| Neurological (4h) | -0.01 (0.02) | 0.65 |
| Neurological (24h) | -0.01 (0.01) | 0.72 |
| Constitutional (4h) | -0.01 (0.01) | 0.48 |
| Constitutional (24h) | 0.00 (0.01) | 0.87 |
| Total (4h) | -0.06 (0.06) | 0.37 |
| Total (24h) | -0.02 (0.03) | 0.53 |

PM_2.5_ was correlated with most of the other components of DE (Additional File 1: Table S3).

**Table S3.** Correlations of PM_2.5_ with other environmental measures in the exposure booth.

| Correlation of PM_2.5_ with: | rho | P value |
| --- | --- | --- |
| NO | 0.65 | <0.00 |
| NO_2_ | 0.57 | <0.00 |
| NOx | 0.66 | <0.00 |
| CO | 0.66 | <0.00 |
| CO_2_ | 0.47 | <0.00 |
| TVOC | 0.78 | <0.00 |
| Total particle number concentration | 0.42 | <0.00 |
| Ultrafine particle number concentration | 0.39 | <0.00 |
| Particle size | 0.50 | <0.00 |
| Temperature | -0.03 | 0.82 |
| Relative humidity | -0.01 | 0.93 |

Abbreviations: CO_2_ = carbon dioxide; CO = carbon monoxide; NO_2_ = nitrogen dioxide; NO = nitrogen oxide; NO_x_ = nitrogen oxides; PM_2.5_ = particulate matter with diameter ≤2.5µm; ppb = parts per billion; ppm = parts per million. TVOC = total volatile organic compounds.


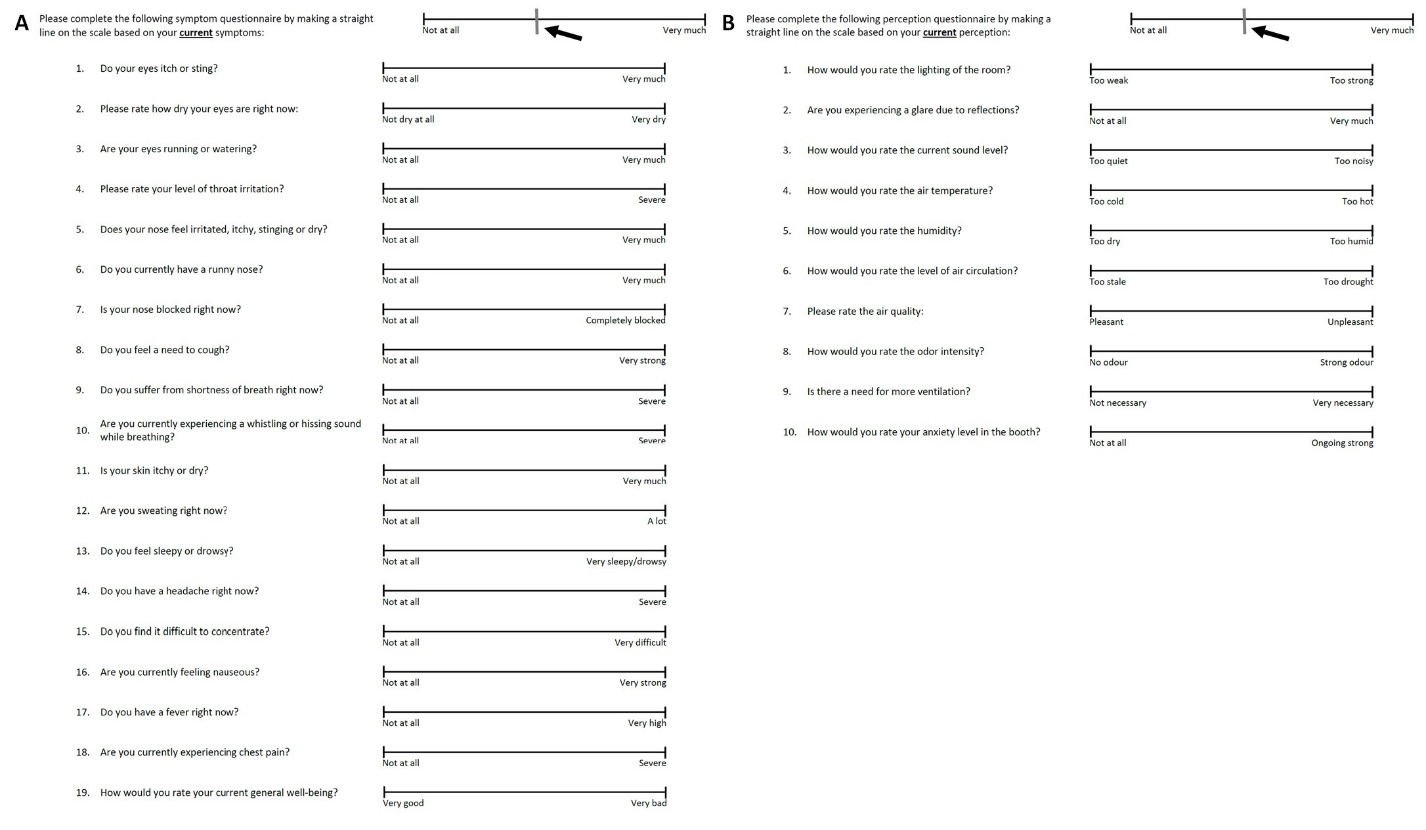


**Figure S1.** Questionnaires. Panel A shows the symptoms visual analog scale questionnaire, while panel B shows the visual analog scale environmental perception questionnaire. Symptom questionnaires were completed by participants before, and at 4h and 24h after the beginning of the exposure. Perception questionnaires were completed in the exposure booth, at the beginning and end of the exposure.


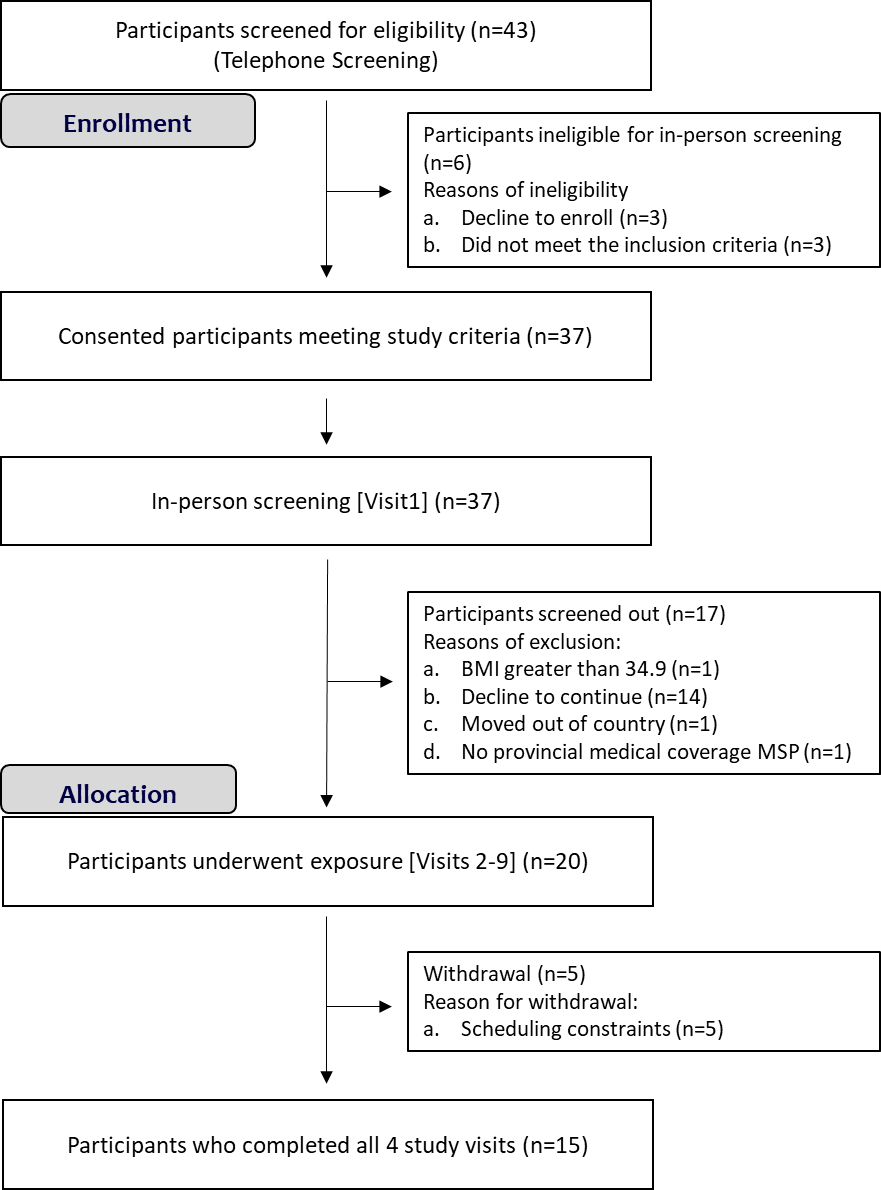


**Figure S2.** Study recruitment flow diagram. Healthy non-smokers aged 19-49 were recruited by referrals and online advertisements into this double-blind crossover exposure study where they were exposed to filtered air and diesel exhaust at 3 concentrations (20, 50, and 150 µg/m^3^ PM_2.5_) for 4 hours, separated by a ≥4-week washout period.
